# Diagnosis and Surgical Management of Pancreatic Insulinoma in a Non-Diabetic Patient: A Case Report

**DOI:** 10.3390/reports8030175

**Published:** 2025-09-08

**Authors:** John Fernando Montenegro, Cristian Eduardo Penagos, Duvy Yasmin Rodríguez, Andres Felipe García-Ramos, Yamil Liscano

**Affiliations:** 1 Grupo de Investigación en Genética, Fisiología y Metabolismo (GEFIME), Ciencias de la Salud Universidad Santiago de Cali, Cali 760035, Colombia; john.montenegro00@usc.edu.co; 2 Programa de Especialización en Medicina Interna, Facultad de Salud, Universidad Santiago de Cali, Cali 760035, Colombia; 3 Departamento de Salud, Facultad de Medicina, Universidad Santiago de Cali, Cali 760035, Colombia; cristian071018@outlook.es; 4 Grupo de Investigación en Salud Integral (GISI), Departamento Facultad de Salud, Universidad Santiago de Cali, Cali 760035, Colombia; duvyrodriguez95@gmail.com; 5 Departamento de Investigación y Educación, Clínica de Occidente S.A., Cali 760046, Colombia; andresfelipegr10@gmail.com

**Keywords:** endogenous hyperinsulinemic hypoglycemia, insulinoma, prolonged fasting test, pancreatic neuroendocrine tumors

## Abstract

**Background and Clinical Significance**: Endogenous hyperinsulinemic hypoglycemia (HHE) is a rare cause of hypoglycemia, primarily associated with insulinomas, the most common functional pancreatic neuroendocrine tumors. This clinical case explores the diagnostic and therapeutic challenges in patients with recurrent hypoglycemia and no history of diabetes, emphasizing the importance of a multidisciplinary approach to optimize outcomes. **Case Presentation:** We present a 45-year-old woman presenting with severe hypoglycemic episodes and persistent neuropsychiatric symptoms for over a year. Prolonged fasting tests, insulin and C-peptide level measurements, and specialized imaging studies (endoscopic ultrasound and pancreatic protocol computed tomography) were conducted. Surgical resection of the identified lesion was subsequently performed. Diagnostic studies confirmed a well-differentiated 2.5 cm insulinoma, treated with partial pancreatoduodenectomy. The patient developed postoperative complications that required interdisciplinary management, ultimately achieving clinical stabilization and sustained normoglycemia. **Conclusions:** This case underscores the importance of considering insulinoma in the differential diagnosis of recurrent hypoglycemia in non-diabetic patients, using Whipple’s triad as a key diagnostic criterion. It also highlights the role played by comprehensive strategies combining functional testing (e.g., prolonged fasting) and advanced imaging to ensure timely treatment and reduce the risk of chronic complications.

## 1. Introduction and Clinical Significance

Endogenous hyperinsulinemic hypoglycemia syndrome (EHHS) is a rare disorder characterized by inappropriate insulin secretion independent of glucose levels, leading to recurrent episodes of severe hypoglycemia [1,2]. This condition is primarily associated with insulinomas (insulin-producing pancreatic neuroendocrine tumors) or, less frequently, β-cell hyperplasia (nesidioblastosis in adults). It manifests with neuroglycopenic and adrenergic symptoms that can compromise quality of life and even patient survival [1,2]. Diagnosis requires confirmation of Whipple’s triad: (1) plasma glucose < 55 mg/dL, (2) hypoglycemia-compatible symptoms, and (3) resolution of symptoms after glucose administration [3,4,5]. Unlike iatrogenic hypoglycemia [5], common in diabetic patients under treatment, EHHS poses a clinical challenge due to its low prevalence, nonspecific presentation, and the need for a rigorous diagnostic evaluation to identify its origin [1].

From an epidemiological perspective, insulinomas, the leading cause of EHHS, have an annual incidence of 1–4 cases per million of the population, accounting for less than 3% of all pancreatic neoplasms and approximately 0.4% of functional pancreatic neuroendocrine tumors [6]. Although 90% of these tumors are benign, solitary, and smaller than 2 cm, their clinical impact is significant due to recurrent hypoglycemic episodes, which may lead to irreversible neurological complications if untreated [1]. Conversely, adult nesidioblastosis, an even rarer entity, presents additional challenges, as its diagnosis requires ruling out insulinomas through histopathological studies [4,7].

The diagnostic approach for EHHS combines biochemical testing (measuring insulin, C-peptide, and proinsulin during hypoglycemia), supervised prolonged fasting tests (the gold standard for confirming endogenous hyperinsulinism), and advanced imaging techniques such as endoscopic ultrasound and pancreatic protocol computed tomography [8,9,10]. However, tumor localization remains challenging in up to 10% of cases, particularly for small or atypical lesions, necessitating multidisciplinary collaboration (endocrinology, radiology, and surgery) to optimize diagnostic accuracy [11,12].

In this context, we present the case of a 45-year-old non-diabetic woman with recurrent severe hypoglycemia (blood glucose ≤40 mg/dL) accompanied by progressive neuropsychiatric symptoms (disorientation, agitation, and transient loss of consciousness). After confirming Whipple’s triad and demonstrating endogenous hyperinsulinism via a 72 h fasting test, a 2.5 cm pancreatic lesion was identified using endoscopic ultrasound.

This patient underwent partial pancreatoduodenectomy, with histopathological confirmation of a well-differentiated insulinoma. Despite postoperative complications (grade B pancreatic fistula), complete symptom resolution and glycemic normalization were achieved through collaborative management with surgery, gastroenterology, and endocrinology teams.

This case highlights three critical aspects of EHHS: (1) the importance of suspecting insulinomas in non-diabetic patients with unexplained hypoglycemia, (2) the utility of structured diagnostic protocols to avoid treatment delays, and (3) the central role played by interdisciplinary collaboration in managing complications and therapeutic decision-making. Additionally, it underscores the need for high-resolution imaging, guided biopsy in ambiguous cases, and long-term surveillance to detect recurrence, particularly in tumors with atypical histological features [3,13].

## 2. Case Presentation

**Figure 1:**
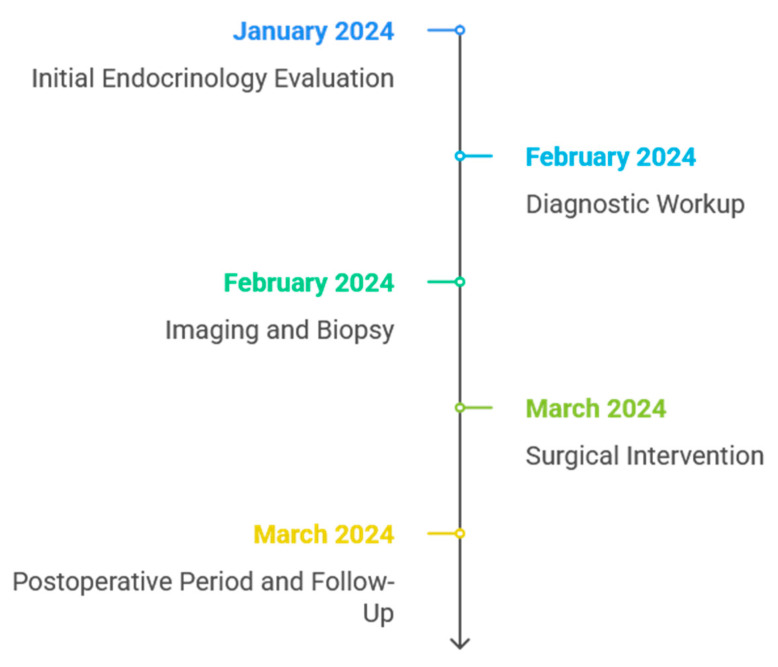
Timeline of clinical events and management of hypoglycemia associated with insulinoma.

Figure 1 illustrates the chronological sequence of key findings and procedures in a 45-year-old female patient with symptomatic hypoglycemia, spanning from emergency admission to insulinoma confirmation and resolution of Whipple’s triad. This timeline outlines initial endocrinology evaluations, laboratory and imaging studies, surgical intervention, and postoperative follow-up, which collectively normalized blood glucose levels and stabilized the clinical course.

### 2.1. Initial Diagnosis and Early Management

A 45-year-old female with no significant medical history presented with a one-year history of disorientation, blurred vision, asthenia, and recurrent hypoglycemia (<40 mg/dL). These findings prompted emergency department admission, where management was initiated with a 10% dextrose bolus.

### 2.2. Endocrinology Evaluation

In January 2024, the endocrinology team confirmed symptomatic hypoglycemia that met Whipple’s triad. Additional tests were ordered, including a 72 h fasting test, insulin, C-peptide, proinsulin, cortisol, and ketone body measurements (see Table 1). Initially, adrenal insufficiency was suspected due to low cortisol levels; however, repeat cortisol and ACTH tests ruled out this diagnosis.

### 2.3. Diagnostic Procedures

By February 2024, ketone bodies showed values of 0 mmol/L, suggesting insulin-mediated hypoglycemia. Despite a prior abdominal MRI (2023) showing no pancreatic lesions, a supervised fasting test was conducted. The results revealed endogenous hyperinsulinemia, with insulin levels of 5.5 µU/mL (reference 2–3 µU/mL) and C-peptide levels of of 2.01 ng/mL (reference: 1.1–1.5 ng/mL) (see Table 1). Subsequent endoscopic ultrasound and octreotide-SPECT scintigraphy were performed to rule out somatostatin receptor-positive tumors or carcinoids, both yielding negative results.

#### ACTH: Adrenocorticotropic Hormone

**Figure 2:**
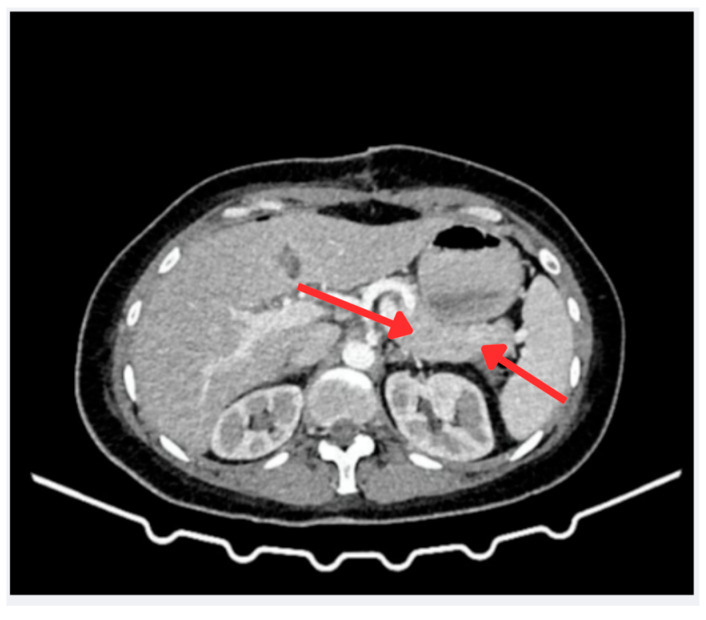
Contrast-enhanced abdominal CT scan (portal phase). Axial view showing the pancreatic region. The red arrows discreetly indicate the area of interest, where no significant abnormalities in density or enhancement are observed in the pancreas or portosplenic region. Based on these imaging findings, no evidence of a neuroendocrine tumor was identified at this stage.

A contrast-enhanced abdominal CT scan with pancreatic protocol (arterial and venous phases) revealed no abnormalities (Figure 2). Finally, endoscopic ultrasound was the modality that identified a hypoechoic lesion in the uncinate process of the pancreas, leading to targeted biopsies.

In the present case, multiple endocrine neoplasia type 1 (MEN1) was ruled out, as no clinical or biochemical abnormalities associated with other typical manifestations of the syndrome, such as primary hyperparathyroidism or pituitary tumors, were found. There was no family history or complementary test findings suggestive of a hereditary condition.

### 2.4. Surgical Intervention and Intraoperative Findings

**Figure 3:**
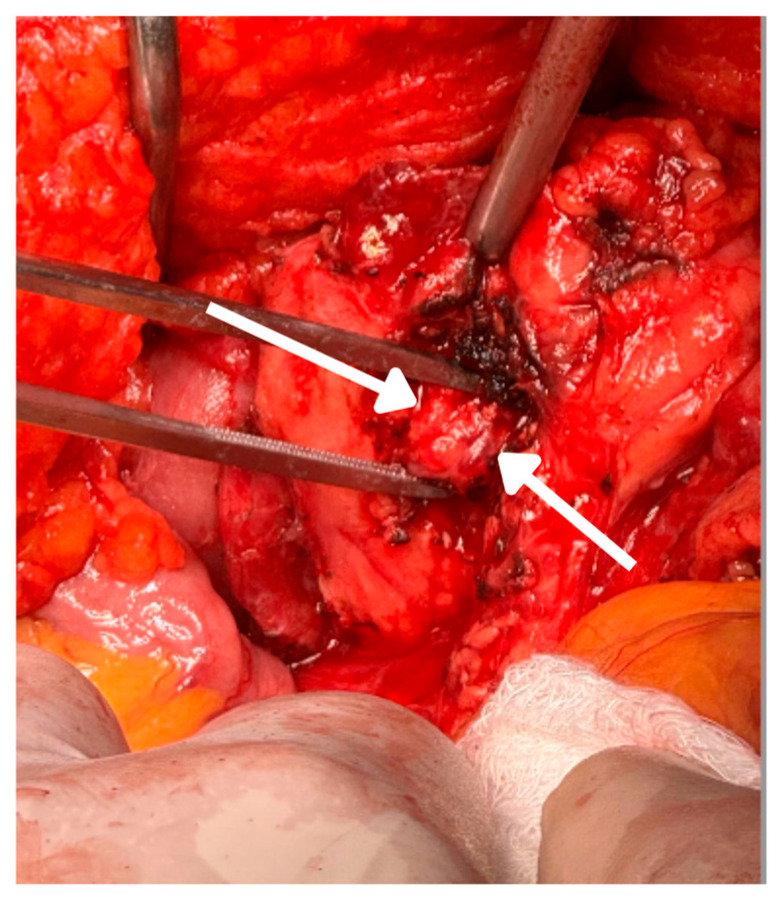
Pancreatoduodenectomy: intraoperative view of the pancreatic uncinate process showing exposed tissue following surgical resection. The arrows discreetly indicate the specific area of the surgical bed corresponding to the resection of an insulinoma. Hyperemic tissue characteristics and cauterized margins are observed, indicating adequate hemostatic control during the procedure. This finding is consistent with pancreatoduodenectomy.

In March 2024, the patient underwent partial pancreatectomy. Intraoperatively, a firm 1 cm nodular lesion was identified on the anterior surface of the pancreatic uncinate process, lateral to the mesenteric vessels. The lesion was resected, and the biopsy of a frozen section confirmed a neuroendocrine tumor (Figure 3).

### 2.5. Postoperative Follow-Up and Clinical Evolution

**Figure 4:**
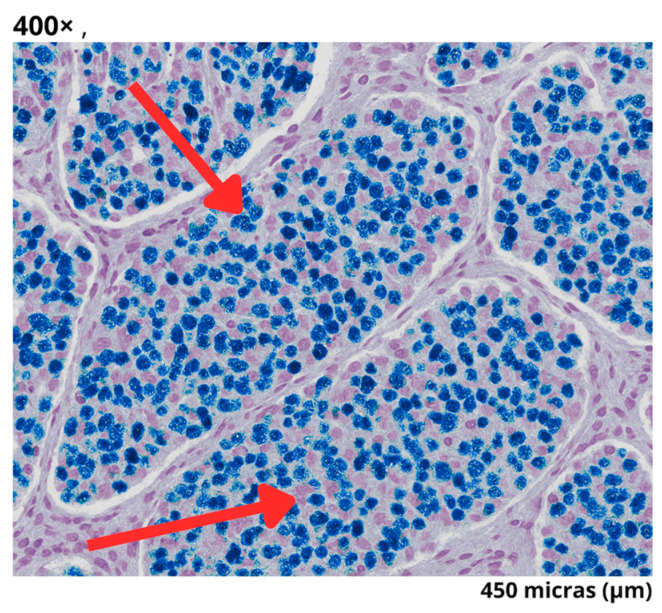
Micrograph of a well-differentiated, grade 3 neuroendocrine tumor. Histological image at 400× magnification (field of view: 450 µm), showing nests of tumor cells separated by fibrous stroma. The red arrows indicate areas with round nuclei and granular chromatin, typical characteristics of this tumor type. The staining reveals a high cellular density, consistent with an elevated Ki-67 proliferation index.

Postoperatively, the patient developed abdominal pain and peritoneal irritation. An abdominal CT scan revealed loculated fluid and gas bubbles near the surgical site, suggestive of a fistula. Following clinical deterioration and fever, a course of piperacillin–tazobactam (4.5 g every 6 h for 10 days) was initiated, and emergency laparotomy identified 1500 cc of hemoperitoneum. The blood cultures remained negative (see Figure 4). The histopathological findings, detailed in Table 2, confirmed the diagnosis of a neuroen-docrine tumor.

**Table 2:** Biopsy results.

Biopsy Type	Findings
Frozen Section	Compatible with neuroendocrine tumor
Pathology	Well-differentiated grade 3 neuroendocrine tumor (2.5 × 1.5 × 1 cm)
Immunohistochemistry/Histology	Well-differentiated neuroendocrine tumor, (2 mitoses per 2 mm^2^) with a Ki-67 proliferation index of 21%

Following infection resolution, the patient was discharged with normalized blood glucose (98–100 mg/dL fasting; 145–160 mg/dL postprandial) and a marked clinical improvement. Outpatient follow-up by gastrointestinal surgery and endocrinology confirmed sustained glycemic control and resolution of Whipple’s triad.

## 3. Discussion

### 3.1. Hypoglycemia in Non-Diabetic Patients and Clinical Relevance

Hypoglycemia in non-diabetic individuals is a rare but potentially dangerous condition, as it can manifest through neuroglycopenic symptoms (e.g., disorientation, blurred vision) and autonomic symptoms (e.g., sweating, palpitations) [14]. In patients meeting Whipple’s triad (hypoglycemic symptoms, documented low glucose, and relief of symptoms after carbohydrate intake), it becomes essential to consider etiologies such as critical illness, medication-related causes, or endocrine disorders, including endogenous hyperinsulinism [15].

### 3.2. Case Presentation and Differential Diagnosis

In the presented case, the patient experienced recurrent symptomatic hypoglycemia with blood glucose readings consistently below 40 mg/dL, despite having no history of diabetes [15]. Initially, adrenal insufficiency was considered due to low cortisol measurements; however, there were no clinical findings to support this diagnosis [16]. The focus subsequently shifted toward endogenous hyperinsulinism after excluding other secondary causes like acute conditions and medications [17]. Management involved the administration of intravenous dextrose and a thorough workup (72 h fasting test, C-peptide, insulin, proinsulin, and anti-insulin receptor antibodies) to rule out insulinoma [16].

### 3.3. Importance of Advanced Diagnostic Techniques

This case underscores the complexity involved in the initial evaluation of refractory hypoglycemia and the necessity for an exhaustive differential diagnosis [18]. Although adrenal insufficiency was initially suspected, extended biochemical studies and hormonal measurements redirected the investigation toward endogenous hyperinsulinism. In addition, invasive methods such as endosonography employing a hepatic vein glucose protocol were crucial in localizing and confirming, via immunohistochemical analysis, a neuroendocrine tumor consistent with an insulinoma; this allows for the correlation of blood insulin levels with the anatomical location of the tumor, increasing diagnostic accuracy in difficult-to-detect insulinomas. Its use is indicated when conventional imaging is negative [18]. These findings enabled early surgical resection, thereby preventing further complications [19]. PET/CT with Ga-68 DOTATATE, as recommended by the NCCN guidelines, is the preferred functional imaging technique for evaluating well-differentiated neuroendocrine tumors (NETs) due to its high accuracy in localizing primary tumors, detecting metastases, and identifying candidates for peptide receptor radionuclide therapy, making it particularly useful in grade 2 and 3 NETs where tumor heterogeneity can be better assessed, especially when combined with FDG PET/CT [2].

### 3.4. Clinical Significance and Comparison with Other Reports

Recognizing rare diseases is crucial because they may be mistaken for more common pathologies or lead to delayed diagnoses [19]. In this context, advanced diagnostic techniques facilitate the precise identification of less frequent causes of hypoglycemia and enable timely treatment [16]. As summarized in Table 3, several authors have reported insulinoma in varying clinical contexts, demonstrating the utility of selective arterial catheterization, supervised fasting tests, and magnetic resonance imaging.

For instance, Saúl E. Torres-Arano et al. (2021) [5] described a 31-year-old male with untreated type 2 diabetes and hypertension who had experienced recurrent hypoglycemic episodes since 2014. Biochemical testing and arterial catheterization revealed elevated insulin levels, leading to laparoscopic distal pancreatectomy, where an insulinoma was confirmed [3]. Similarly, Chenyang Zhang et al. (2022) [4] reported a 79-year-old male with severe hypoglycemia (blood glucose of 1.01 mmol/L) and a 0.8 cm insulinoma in the pancreatic tail, identified through contrast-enhanced computed tomography. After surgical resection, the patient’s blood glucose levels normalized without recurrence. Another report by Zhang et al. (2022) [4] detailed insulin autoimmune syndrome (IAS) in a 68-year-old male with type 2 diabetes, which improved with prednisone and acarbose. Meanwhile, Kiran Shah et al. (2021) [3] described a 45-year-old female experiencing hypoglycemic episodes alleviated by sugar ingestion; further evaluation confirmed endogenous hyperinsulinism, and the condition resolved following surgical resection.

### 3.5. Prognostic Implications in Diagnosis and a High Ki-67 Index

Although insulinomas are typically well-differentiated neuroendocrine tumors with low proliferative activity, our case revealed a Ki-67 index of 21%, which places it at the upper limit for grade 3 tumors according to the WHO classification and NCCN Guidelines for Neuroendocrine and Adrenal Tumors [20,21]. This suggests potential tumor heterogeneity and a higher risk of recurrence or aggressive behavior. Hofland et al. emphasize that insulinomas with Ki-67 > 10% may show reduced response to surgical resection alone, highlighting the need for close follow-up and possible adjuvant strategies [6,21].

Despite negative findings on MRI and CT, endoscopic ultrasound (EUS) successfully identified a hypoechoic lesion in the pancreatic uncinate process, guiding targeted biopsy and resection. This aligns with prior studies reporting EUS sensitivity of up to 94% for occult insulinomas, particularly for lesions <1.5 cm [11,12,22]. Early use of EUS in the diagnostic algorithm can prevent delays and reduce the risk of neuroglycopenic complications [8,23].

### 3.6. Multidisciplinary Approach and the Value of Early Suspicion

The case discussed aligns with these reports, as the patient had no significant medical history yet presented with refractory hypoglycemia lacking any evident secondary causes, ultimately leading to an insulinoma diagnosis. A comprehensive approach, guided by endocrinology and supported by imaging and immunohistochemical studies, enabled an effective and timely intervention that reduced morbidity and mortality [22]. Alongside laboratory findings, clinical acumen and the proper interpretation of advanced diagnostic tests proved pivotal in detecting this neuroendocrine tumor [14].

Endoscopic ultrasound is essential for assessing the tumor’s proximity to the pancreatic duct and guiding surgical resectability. Laparoscopic or robot-assisted enucleation is feasible when the lesion is identified preoperatively by CT or EUS, and there is a safe distance from the pancreatic or bile duct. Insulinomas located in the pancreatic head rarely require pancreatoduodenectomy, thus significantly reducing morbidity [21]. In patients who are not candidates for surgery, chemical ablation with ethanol or other sclerosing agents represents a valid therapeutic alternative, guided by endoscopic ultrasound to ensure precise localization, thereby optimizing the safety and efficacy of the treatment by delivering the ablative agent directly to the lesion [22,23].

### 3.7. Limitations and Recommendations

One of the main challenges in identifying hypoglycemia in non-diabetic patients is the variability and subtlety of neuroglycopenic symptoms, which can postpone diagnosis and potentially lead to incorrect interpretations [17]. Furthermore, the availability of advanced diagnostic methods, such as endosonography or selective arterial catheterization, may vary among medical centers, complicating the early confirmation of suspected cases. Maintaining a high index of clinical suspicion is therefore recommended for patients experiencing recurrent hypoglycemic episodes without clear causes, alongside an early multidisciplinary approach involving endocrinologists, surgeons, and radiologists [13].

An additional limitation of this case is the use of octreotide-SPECT scintigraphy (Octreoscan), which is considered outdated compared to PET/CT with Ga-68 DOTATATE. According to the NCCN guidelines, Ga-68 PET/CT is the preferred imaging modality for well-differentiated neuroendocrine tumors (NETs) due to its higher sensitivity to detect both primary and metastatic lesions. However, although Octreoscan is outdated, it still demonstrates acceptable sensitivity for identifying lesions in well-differentiated NETs, especially in centers where Ga-68 PET/CT is not available. Recent studies support its diagnostic value, albeit with limitations compared to modern techniques [24,25].

Standardized protocols for performing and interpreting prolonged fasting tests and imaging studies can optimize the diagnosis of insulinoma or other uncommon etiologies of hypoglycemia. Finally, timely referral to specialized centers is critical for ensuring comprehensive care and minimizing the complications associated with these rare pathologies [26].

## 4. Conclusions

This case of EHHS secondary to pancreatic insulinoma highlights the diagnostic and therapeutic complexities inherent in rare endocrine disorders. The patient’s presentation with recurrent hypoglycemia and neuropsychiatric symptoms, despite lacking diabetes or other predisposing factors, exemplifies the critical need to consider insulinoma in the differential diagnosis of non-diabetic hypoglycemia. The successful resolution of her condition relied on adherence to Whipple’s triad, systematic biochemical evaluation, and advanced imaging techniques, including endoscopic ultrasound and contrast-enhanced MRI, which localized the lesion despite initial inconclusive studies.

The multidisciplinary collaboration among endocrinology, radiology, gastroenterology, and surgical teams was pivotal in navigating postoperative complications (e.g., pancreatic fistula) and achieving sustained normoglycemia. This aligns with the existing literature emphasizing the role played by structured protocols—prolonged fasting tests, targeted imaging, and histopathological confirmation—in optimizing outcomes. Notably, this case mirrors challenges reported elsewhere, such as delayed diagnosis due to subtle neuroglycopenic symptoms and variability in access to specialized diagnostics.

## Figures and Tables

**Table 1:** Laboratory and imaging studies conducted during hospitalization.

Test	Result	Reference Range
Ketone Bodies	0.2 mmol/L	0–1 mmol/L
Serum Cortisol 1	4.70 µg/dL	5.27–22.45 µg/dL
C-Peptide	2.01 ng/mL	1.1–1.5 ng/mL
Serum Cortisol 2	13.35 µg/dL	5.27–22.45 µg/dL
ACTH	9.2 pg/mL	4.7–48.8 pg/mL
Insulin Levels	5.5 µU/mL	2–3 µU/mL
Serial Blood Cultures	Negative	-
72 h Fasting Test	45–59–148–49–55–64–60 mg/dL	60–100 mg/dL

**Table 3:** Clinical case reports.

Author	Age and Gender	Medical History	Clinical Manifestation	Procedure Performed	Treatment
Saúl E. Torres-Arano et al. (2021) [5]	31-year-old male	Untreated type 2 diabetes mellitus, hypertension, and depression	Symptoms of diaphoresis, palpitations, malaise, and syncope, with capillary glucose of 55 mg/dL, improving with glucose administration	Biochemical tests and selective arterial catheterization, revealing elevated insulin levels	Surgical resection
Chenyang Zhang et al. (2022) [4]	79-year-old male	N/A	Severe hypoglycemia (blood glucose of 1.01 mmol/L)	IV contrast tomography	IV insulin, surgical resection
Chenyang Zhang et al. (2022) [4]	68-year-old male	Type 2 diabetes	Recurrent hypoglycemia with glucose levels between 2.8 and 21.6 mmol/L	Serum glucose, positive autoimmune antibodies (anti-insulin)	Prednisone and acarbose for 10 months
Kiran Shah et al. (2021) [3]	45-year-old female	N/A	Restlessness, anxiety, palpitations, excessive sweating, dizziness, and tremors, relieved by sugar intake	Supervised 72 h fast, elevated insulin, proinsulin, and C-peptide levels during critical samples	Surgical resection

N/A: not applicable; IV: intravenous.

## Data Availability

The original contributions presented in this study are included in the article. Further inquiries can be directed to the corresponding author.

## Abbreviations

The following abbreviations are used in this manuscript:

EHHS	Endogenous Hyperinsulinemic Hypoglycemia Syndrome
ACTH	Adrenocorticotropic Hormone
IAS	Insulin Autoimmune Syndrome
FDG	Fluorodeoxyglucose
FDG PET/CT	Fluorodeoxyglucose Positron Emission Tomography/Computed Tomography
MEN1	Multiple Endocrine Neoplasia Type 1
EUS	Ultrasonography
